# Determinants of the behavioral intention to use a mobile nursing application by nurses in China

**DOI:** 10.1186/s12913-021-06244-3

**Published:** 2021-03-12

**Authors:** Minghao Pan, Wei Gao

**Affiliations:** 1 Medical College, Xinyang Normal University, Xinyang, China; 2 PICC Outpatient, Qilu Hospital of Shandong University, Jinan, China

**Keywords:** Health technology, Behavioral intention, Mobile applications, Nursing

## Abstract

**Background:**

Although a mobile nursing application has began to adopt in nursing, few studies have focused on nurses’ behavioral intention of it. The objective of this study is to gain insight into the behavioral intention of nurses, i.e. chinese nurses of the future, to use a mobile nursing application. This study adopted an extension of the Unified Theory of Acceptance and Use of Technology to examine Chinese nurses’ acceptance of a mobile nursing application.

**Methods:**

A total of 1207 nurses participated in the cross-sectional survey. The majority of nurses were female (96.2%). The mean age of the participants was 34.18 (SD 7.39). The hypothesized relationships were tested using AMOS structural equation model.

**Results:**

All constructs exhibited an acceptable level of reliability and validity with Cα and CR > 0.7 and AVE > 0.5. An extension of the Unified Theory of Acceptance and Use of Technology Model had good explanatory power for nurses’ behavioral intention of a mobile nursing application. Although effort expectancy and perceived risks had a surprisingly insignificant effect on nurses’ behavioral intention to use a mobile nursing application, performance expectancy, social influence, facilitating conditions, self-efficacy, and perceived incentives demonstrated significant influence with β = .259, *p* < .001, β = .296, *p* < .001, β = .063, *p* = .037*,* β = .344, *p* < .001*,* β = .091, *p* = .001*,* respectively.

**Conclusion:**

With 70.2% of the variance in behavioral intention to use a mobile nursing app explained by this model, it could be helpful for potential adopters, and further investigation should test the actual usage behavior for a mobile nursing app and investigate the related factors.

**Supplementary Information:**

The online version contains supplementary material available at 10.1186/s12913-021-06244-3.

## Background

In China,nursing informationization refers to the combination of traditional nursing work and information technology in order to improve the comprehensive nursing quality and accelerate the modernization process. With the development of nursing informatization, nursing quality is effectively improved [1]. Owing to a large number of patients in China, it is very necessary to develop nursing informatization to insure nursing quality. As of August 2018, the number of Chinese mobile netizens had reached 788 million [2]. Meanwhile, according to the National Nursing Development Plan (2016–2020) in China, nursing informatization should be vigorously promoted with the rapid development of information technologies such as big data, cloud computing, the Internet of Things and mobile communications [3]. Thanks to the Chinese government’s policy in nursing informatization, nursing clinical practice has changed largely in these years. One of the most important changes is that few Chinese nurses begin to try to use a mobile nursing application (app) in nursing care [4].

A mobile nursing app is a type of mobile app that assists nurses with clinical nursing work. A mobile nursing app could help nurses in conducting nursing evaluations of patients by integrating the electronic medical record system and a wireless local area network [5]. In addition, a mobile nursing app served as a new communication channel for nurses and patients [4]. Moreover, through the use of a mobile nursing app nurses could deliver cost-effective rehabilitation guidance to patients [6–8]. A mobile nursing app in nursing services is still in its infancy. Previous studies mainly focus on the construction of a mobile nursing app [9, 10], the investigation of application needs [11, 12] and the exploration of the feasibility of nursing [13–15]. A mobile nursing app has the characteristics by the use of mobile applications, such as the emphasis on the individual’s smart phone use ability [16], and the ability to accept mobile applications [17], which puts forward higher requirements for nurses. Although the Chinese government has relevant documents supporting nursing informatization, there is no mandatory policy on nurses’ use of a mobile nursing app. Therefore, nurses’ intention to use a mobile nursing app and related influencing factors need to be paid more attention to.

Since the 1980s, individual behavior and behavioral intention of information technology have become a research hotspot in the field of information systems, and a series of information technology acceptance models have been formed [18–20]. Among information technology accptance models, the Technology Acceptance Model (TAM) and the Unified Theory of Acceptance and Use of Technology (UTAUT) are the most commonly used [21, 22]. Meanwhile, the UTAUT model has stronger explanatory power than TAM in terms of explanation [21]. A number of models have been used to predict factors associated with the behavioral intention of m-Health [23–27]. Recently, UTAUT is considered as the most valid technology acceptance model used to assess behavioral intention and actual use behavior of m-Health [28–32]. Previous studies have shown that users’ behavioral intention of information technology is the basic premise of users’ use behavior [33, 34]. In previous studies of nurses’ behavioral intention of a mobile app, scholars highlighted the necessity of understanding nurses’ behavioral intention of a mobile app [35–37]. In China, some studies have investigated the behavioral intention to use a mobile nursing application by patients [16, 38]; however, few studies investigated the behavioral intention to use a mobile nursing app by Chinese nurses. Therefore, the purpose of this study was to:

1. Introduce an modified theoretical model constructed based on the Unified Theory of Acceptance and Use of Technology (UTAUT) model.

2. To what extent is the behavioral intention to use a mobile nursing application related to the factors from an extension of the Unified Theory of Acceptance and Use of Technology (UTAUT) model.

### Theoretical background and hypotheses

UTAUT is widely used to predict users’ intention and behavior, explaining 69% of the variance in users’ behavioral intention (BI) [20]. UTAUT considers the decisive effect of four factors—performance expectancy (PE), effort expectancy (EE), social influence (SI), and facilitating conditions (FC) —on users’ behavioral intention. The three main viewpoints of UTAUT are as follows: firstly, behavioral intention is directly influenced by performance expectancy, effort expectancy and social influence; secondly, use behavior depends on the direct influence of facilitating conditions and behavioral intention; thirdly, age, gender, experience, and voluntariness of use mediate the impact of performance expectancy, effort expectancy and facilitating conditions on behavioral intention [20].

A number of previous studies used UTAUT model to explore the intention of patients and medical staff to use m-Health [39–42]. Hoque et al. [39] explored the influencing factors of elderly patients’ intention to use m-Health in Bangladesh based on UTAUT, and found that elderly patients’ intention to use m-Health was affected by performance expectancy, effort expectancy and social influence, but not by facilitating conditions. Hsieh et al. [40] pointed out that UTAUT could better explain patients’ intention to use the personal health system. Kim et al. [41] explored the influencing factors of 942 health care workers’ intention to use electronic health record systems through questionnaires and system log files. The results showed that the intention to use the electronic health record system was influenced by the performance expectancy. Through literature review, Hennemann et al. [42] pointed out that it was necessary to discuss medical staff’s intention to use m-Health based on UTAUT, and the research results showed that performance expectancy and social influence positively affected medical staff’s intention to use m-Health.

The proposed model established in this study hypothesizes that apart from the four basic constructs in UTAUT, perceived risk (PR), self-efficacy (SE), and perceived incentives (PI) may have significant loading on the behavioral intention in China. The extension on the model is to increase the prediction of the intention to use a mobile nursing app by nurses. The following sections explain the hypotheses developed in this study for investigating the research question.

#### Performance expectancy

It is defined as the degree to which a person believes that using certain information technology can enhance work performance [20]. Therefore, the variable of performance expectancy in our study is defined as a nurse believes that using a mobile nursing app can enhance work performance. A mobile nursing app as a new information technology and new nursing mode, it will be more willing to use when nurses perceive that it was more helpful to nursing. Consequently, we proposed the following hypothesis:

H1: Performance expectancy will be positively associated with nurses’ behavioral intention.

#### Effort expectancy

It is defined as the degree of ease associated with the use of certain information technology [20]. Therefore, the variable of effort expectancy in our study is defined as the degree of ease associated with the use of a mobile nursing app by a nurse. The less effort nurses need to invest, the more likely they are to accept a mobile nursing app over time. Consequently, we proposed the following hypothesis:

H2: Effort expectancy will be positively associated with nurses’ behavioral intention.

#### Social influence

It is defined as the degree to which a person perceives that important others believe he or she should use certain information technology [20]. Therefore, the variable of social influence in our study is defined as a nurse perceives that important others believe he or she should use a mobile nursing app. A mobile nursing app will be more willing to use when nurses receivd the more positive information about it. Consequently, we proposed the following hypothesis:

H3: Social influence will be positively associated with nurses’ behavioral intention.

#### Facilitating conditions

It is defined as the degree to which a person believes that infrastructure supports the use of certain information technology [20]. Reviewing the literature on the behavioral intention of information technology, facilitating conditions has an effect on behavioral intention [23, 41, 43]. The more sufficient application support (such as application operation response, application page layout, etc.) and technical support conditions (such as application use training) that nurses perceive when using a mobile nursing app, the more likely they are to use. Consequently, we proposed the following hypothesis:

H4: Facilitating conditions will be positively associated with nurses’ behavioral intention.

#### Perceived risk

It is defined as the degree to which a person’s judgment that using certain information technology may produce bad results [44]. Issues related to perceived risk pose a obstacle to the adoption of m-Health [45, 46]. The increase of perceived risk could directly reduce person’s behavioral intention to use m-Health [47]. Hsieh et al. [48] studied medical workers’ willingness to use health cloud technology and pointed out that perceived risk impacted negatively on their behavioral intention. Kim et al. [41] showed that patients’ perceived risk of health information technology negatively affected their intention to use health information technology. Whether mobile apps are secure or not is related to nurses’ psychological cognition [47]. Therefore, the variable of perceived risk in our study is defined as a nurse’s judgment that using a mobile nursing app may produce bad results. Previous studies have pointed out that perceived risk negatively affects individuals’ intention to use m-Health [40, 47, 49]. At the same time, mobile applications may disclose users’ personal information, which brings some risks to users [26]. Nurses may perceive that the use of a mobile nursing app will harm their own interests, such as binding software, privacy disclosure, etc., which may lead to a decrease in the intention of nurses to use a mobile nursing app. Therefore, we proposed the following hypothesis:

H5: Perceived risk will be negatively associated with nurses’ behavioral intention.

#### Self-efficacy

In 1977, Bandura [50] proposed the concept of self-efficacy based on social cognition theory. In the research field of information technology, Davis [51] discussed for the first time the influence of self-efficacy on the intention to use information technology, and pointed out that self-efficacy was an important factor affecting individuals’ intention to use information technology. Self-efficacy is defined as the degree to which a person’s perceived ability to use certain information technology [52]. It is an important determinant of users’ perceptions [46, 53–55]. Therefore, in the context of our research, self-efficacy is defined as a nurse’s perceived ability to acquire expected information from mobile apps. Chung et al. [56] showed that the self-efficacy affected the behavioral intention of nurses on patient personal health records. Yamin et al. [57] used an extension of the UTAUT model and self-efficacy to explore users’ intention to use wireless sensor network application for medical assistance. As a type of mobile application, a mobile nursing app emphasizes the nurse’s ability to operate mobile applications. Therefore, the more self-efficacy nurses perceive their mobile applications, the more likely they are to use a mobile nursing app. We proposed the following hypothesis:

H6: Self-efficacy will be positively associated with nurses’ behavioral intention.

#### Perceived incentives

It is defined as the degree to which a person believes that they should be rewarded for his or her behavior [58]. In the field of m-Health, financial incentives were shown to be positive reinforcements of behavior [59]. Moreover, Rho, Choi, and Lee [60] found that Perceived incentives influenced physicians’ intention to use m-Health [61]. A mobile nursing app is a new technology and it requires nurses to spend extra time and energy to adapt [62]. Therefore, it is very necessary to understand nurses’ views on the incentive mechanism of a mobile nursing app. A mobile nursing app is a service innovation mode under nursing informatization. The higher the compensation that clinical nurses perceive from using a mobile nursing app, the more likely nurses willing to use a mobile nursing app. Therefore, we proposed the following hypothesis:

H7: Perceived incentives will be positively associated with nurses’ behavioral intention.

**Fig. 1:**
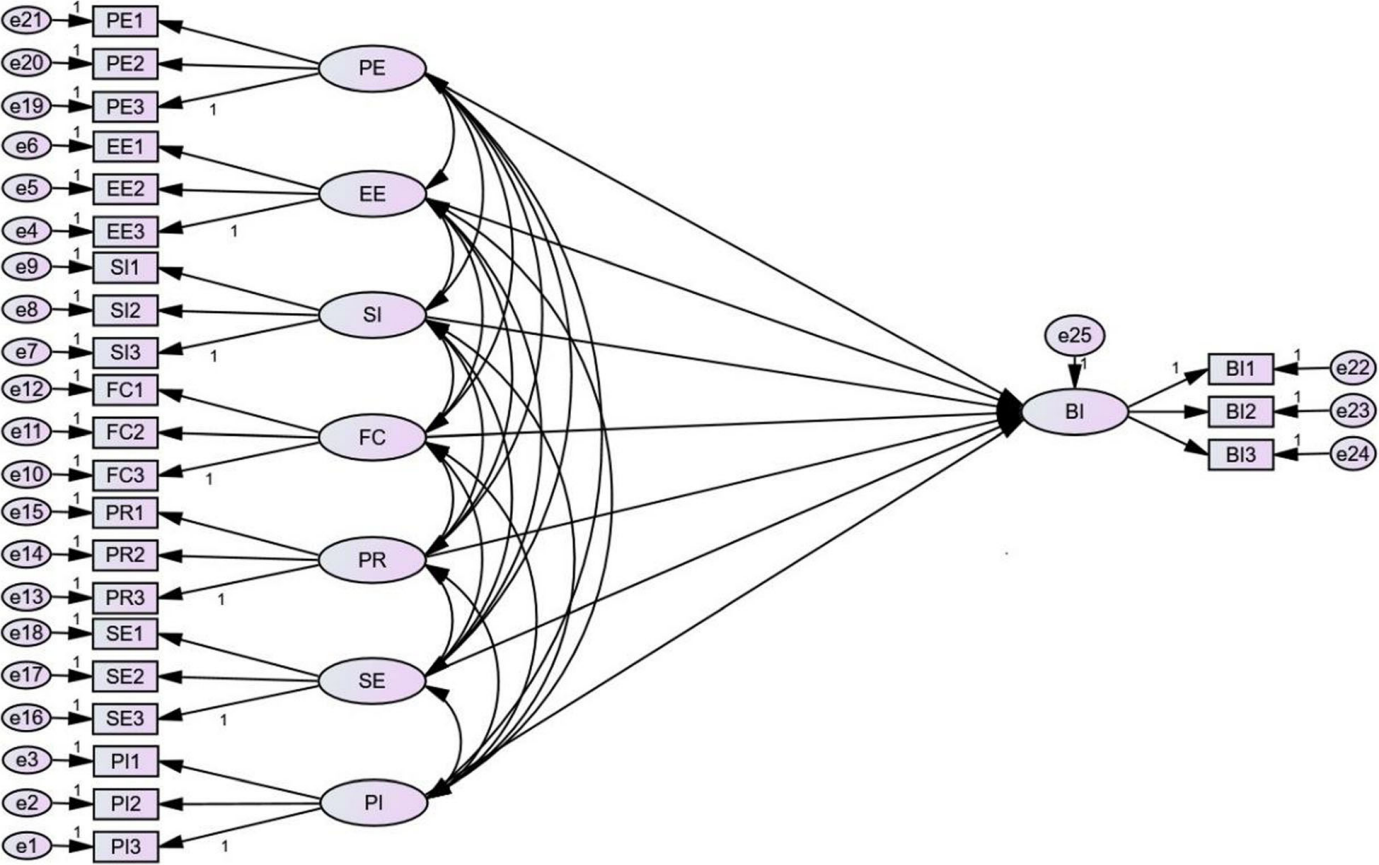
The influence factor model of nurses’ behavioral intention to use a mobile nursing app

The framework of our study was constructed by primarily referring to related theoretical ideas to study the impact of nurses’ behavioral intention to use a mobile nursing app (Fig. 1).

## Methods

### Participants

In this study, participants were recruited by means of convenient sampling. We sent questionnaires to nursing managers in seven hospitals and entrusted them to disseminate the research questionnaire in the working contact group for hospital nurses. Participants completed a survey hosted through the online survey platform Wenjuanxing. Eligible nurses must: (1) have a Registered Nurse license; (2) have worked for at least 1 year; and (3) currently work full-time. Exclusion criteria were currently having taken extended sick leave or maternity leave (more than 3 months). Kendall’s sample estimation method was adopted, and the sample size was 10 ~ 20 times of the number of questionnaire items. There were 31 items in this questionnaire, and considering the unqualified rate of 20%, the sample content of this questionnaire was set at 744.

### Questionnaire

The questionnaire was made up of two parts. The first part of the questionnaire captured demographic information of nurses. Nurses were asked about their age, gender, seniority, work experience of nursing, nurse manager or not, monthly income and qualification.

The second part of the questionnaire was that the questions on nurses’ behavioral intention and influencing factors of a mobile nursing app. The questions on nurses’ behavioral intention and influencing factors of a mobile nursing app have eight research variables. In order to ensure the item accurate reliable measurements of research variables, our study, firstly, based on the previous research results on influencing factors of m-Health behavioral intention, the maturity measurement items related to the study variables were sorted out. The items concerning UTAUT were adapted from Venkatesh et al. [20]; the items of perceived risk were adapted from Cox et al. [44]; the items of self-efficacy were adapted from Compeau et al. [52]; and the items of perceived incentives were adapted from Rho et al. [60]. Sencondly, combined with the situation of nurses using a mobile nursing app, the questions on nurses’ behavioral intention and influencing factors of a mobile nursing app were adapted to the situation. All the questions of this part were measured using a five-point Likert scale, from strongly disagree (score = 1) to strongly agree (score = 5).

### Quality control

The online questionnaire released by the Wenjuanxing platform can only be submitted successfully after all questions have been answered, and each mobile device can only submit the questionnaire once. Therefore, there were no missing values in the collected questionnaire data. And it would not happen that one person submitted the questionnaire twice. Questionnaire data collected by the Wenjuanxing platform can be directly exported as Excel text. Therefore, the questionnaire data did not need manual data entry, which could effectively avoid data entry errors.

In order to ensure that the sample data of analysis had the correct filling quality, all questionnaires were screened in three steps. Firstly, through the data derived from the Wenjuanxing platform, the questionnaire with IP address of Shandong Province was selected. Secondly, questionnaires that took less than 2 min to complete were excluded. Finally, eliminate the questionnaire with no difference in answers.

### Ethical considerations

This study conformed to the principles outlined in the Declaration of Helsinki. Ethical approval was obtained from the Medical Ethics Committee of Shandong University (code: 2018-R-017). The purpose of this study was explained at the beginning of the electronic questionnaire. Nurses completed the questionnaires after clicking the informed consent button. All survey responses were treated confidentially.

### Data analysis

SPSS 19.0 (SPSS; IBM, Armonk, NY, USA) and AMOS 21.0 (SPSS; IBM, Armonk, NY, USA) were used for analyses. Firstly, descriptive analysis was performed to describe characteristics of participants by using SPSS 19.0. Secondly, in measurement model assessment, reliability and validity of items were determined using Cronbach alpha (α), standardized factor loading, composite reliability (CR) and average variance extracted (AVE) to check for both reliability and validity of constructs by using SPSS 19.0 and AMOS 21.0. Thirdly, AMOS 21.0 was applied to build a structural equation model for influencing factors of nurses’ behavioral intention to use a mobile nursing app. There were 8 latent variables and 24 observed variables in this structural equation model. AMOS 21.0 was further applied to test the structural equation model fitting and hypothesis test of influencing factors of nurses’ behavioral intention to use a mobile nursing app.

## Results

### Demographic information

In total, 1207 nurses participated in this study (response 96.71%; 1161 femal and 46 male). The mean age of the participants was 34.18 (SD 7.39). 233 (18.48%) of the respondents were aged less than 28 years, 503 (41.49%) were aged 28 to 35 years, 330 (27.34%) were aged 36 to 43 years, 136 (11.27%) were aged 44 to 51 years, and 16 (1.33%) were aged more than 51 years. 538 (44.57%) of the nurses had less than 10 years in work experience, 367 (30.41%) had 10 to 19 years in work experience, 255 (21.13%) had 20 to 29 years in work experience, and 47 (3.89%) had more than 29 years in work experience.

One fifth (27.59%) of the respondents were nurse manager and four fifth (72.41%) were not. 5.63% of the respondents had less than 2000 yuan in monthly income, 26.01% of the respondents had 2000 to 3999 yuan in monthly income, 34.71% had 4000 to 5999 yuan in monthly income, 21.04% had 6000 to 7999 yuan in monthly income, and 12.6 had higher than 8000 yuan in monthly income. 15.33% of the respondents had college degree, 81.77% of the respondents had bachelor degree, and 2.9% of the respondents had master degree.

### Average score of variables

The results showed that the average score of nurses’ behavioral intention to use a mobile nursing app was 4.13 ± 0.85. The average score of performance expectancy was 4.33 ± 0.78. The average score of effort expectancy was 3.92 ± 0.86. The average score of social influence was 4.03 ± 0.82. The average score of facilitating conditions was 4.38 ± 0.75.

The average score of perceived risk was 3.42 ± 1.02. The average score of self-efficacy was 3.91 ± 0.84. The average score of perceived incentives was 3.71 ± 1.14. The results of normal test showed that all the variables obey normal distribution.

### Measurement model assessment

Reliability analysis was to test the stability and consistency of questionnaire measurement results [63]. In this study, Cronbach’s α coefficients was used to measure the internal consistency of the variables. Cronbach’s α coefficients of performance expectancy, effort expectancy, social influence, facilitating conditions, perceived risk, self-efficacy, perceived incentives and behavioral intention were .919, .921, .908, .937, .880, .887, .881 and .955, respectively, which met the standard of Cronbach’s α coefficient greater than 0.7 [63] (Table 1).

**Table 1:** Reliability and convergent Validity of the measurement model

Variable	Items	Loading	*P*	Cronbach alpha α	Composite reliability
Performance Expectancy	PE1	.850	< .001	.919	.920
	PE2	.91	< .001		
	PE3	.912	< .001		
Effort Expectancy	EE1	.931	< .001	.921	.926
	EE2	.953	< .001		
	EE3	.806	< .001		
Social Influence	SI1	.796	< .001	.908	.912
	SI2	.937	< .001		
	SI3	.903	< .001		
Facilitating Conditions	FC1	.894	< .001	.937	.937
	FC2	.950	< .001		
	FC3	.894	< .001		
Perceived Risk	PR1	.861	< .001	.880	.882
	PR2	.890	< .001		
	PR3	.782	< .001		
Self-efficacy	SE1	.828	< .001	.887	.893
	SE2	.924	< .001		
	SE3	.819	< .001		
Perceived Incentives	PI1	.837	< .001	.881	.881
	PI2	.869	< .001		
	PI3	.826	< .001		
Behavioral Intention	BI1	.910	< .001	.955	.956
	BI2	.948	< .001		
	BI3	.954	< .001		

Convergent validity was comprehensively measured by three indicators, including standardized factor loading, average variance extracted (AVE) and composite reliability (CR) [64, 65]. The lowest value of standardized factor loading of each item was .782, greater than 0.5 [64]. The lowest value of composite reliability (CR) in each variable was .881, greater than 0.7 [64, 65]. AVE value of each variable ranged from .713 to .879, greater than 0.5 [64, 65] (Table 1 and Table 2).

**Table 2:** Discriminant validity of measurement model

Constructs	AVE	PE	EE	SI	FC	PR	SE	PI	BI
Performance Expectancy	0.794	**0.891**							
Effort Expectancy	0.808	0.599	**0.899**						
Social Influence	0.776	0.733	0.743	**0.881**					
Facilitating Conditions	0.834	0.664	0.596	0.742	**0.913**				
Perceived Risk	0.715	0.012	0.035	0.048	0.135	**0.856**			
Self-efficacy	0.737	0.536	0.704	0.634	0.566	0.112	**0.858**		
Perceived Incentives	0.713	0.073	0.022	0.037	0.026	0.679	0.063	**0.844**	
Behavioral Intention	0.879	0.711	0.660	0.757	0.649	0.006	0.702	0.099	**0.938**

Off-diagonal elements are correlations among constructs. The bold fonts in the leading diagonals are the square roots of AVEs

**Fig. 2:**
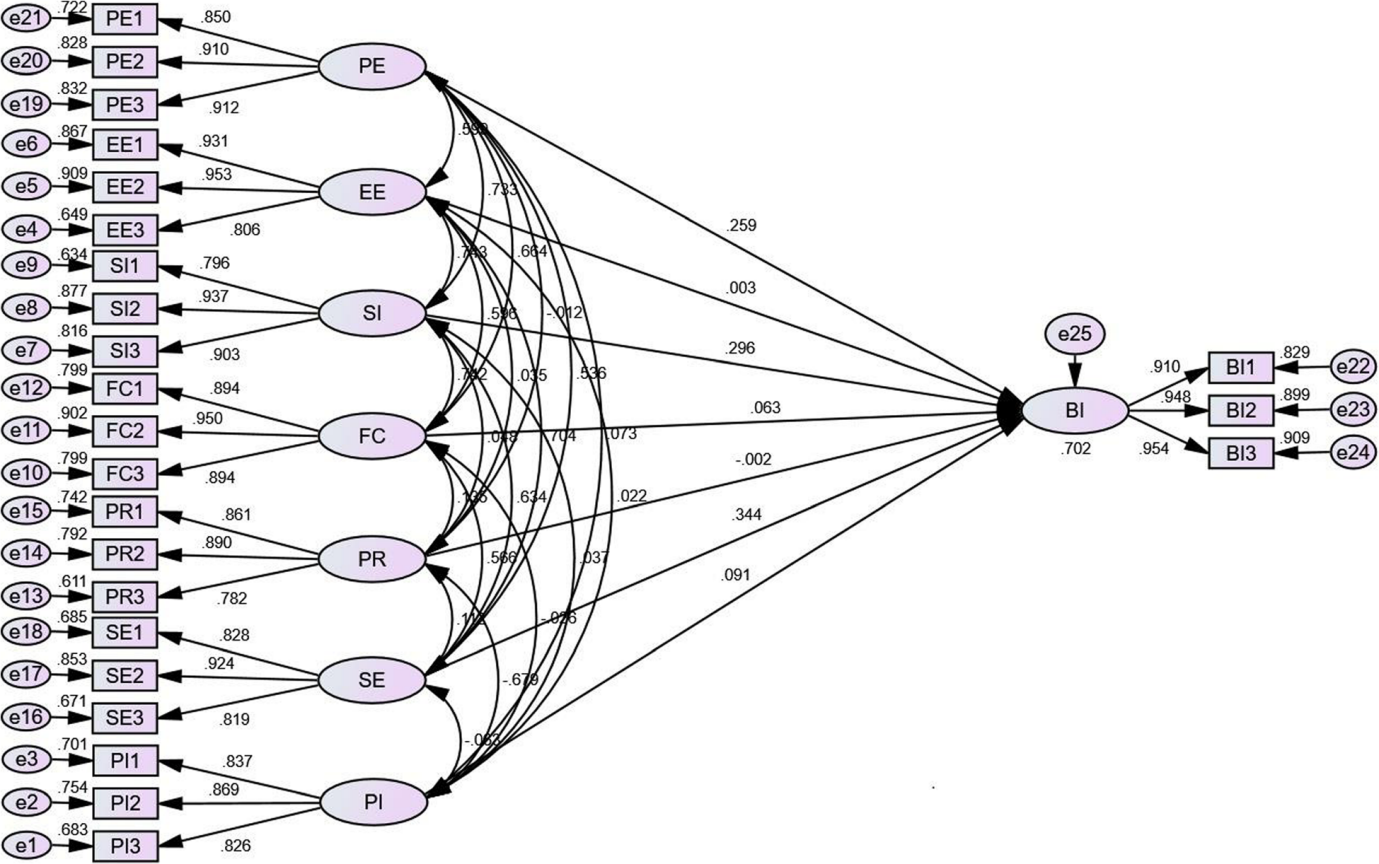
The results of the influence factor model of nurses’ behavioral intention to use a mobile nursing app

Discriminant validity specifies the degree to which each construct measures different variables of the study. We assessed the constructs discriminant validity by comparing the square roots of average variance extracted (AVE) to the absolute values of correlation between constructs [66]. As presented in Table 2, all of the square roots of AVEs (diagonal) are greater than the correlations among constructs, which illustrates the discriminant validity (Table 2 and Fig. 2).

### Structural model assessment

Overall, the values of fit statistics of the structural model indicated a good model fit: GFI = 0.934, AGFI = 0.911, NNFI = 0.963, CFI = 0.971, IFI = 0.971, and RMSEA = 0.054, and χ^2^/df = 4.540. The model explained 70.2% of the variance in behavioral intention, which indicates an excellent *R*^2^ [65].

We found that performance expectancy, social influence, facilitating condition, self-efficacy and perceived incentives had a positive significant effect on nurses’ behavioral intention to use a mobile nursing app. Thus, H1, H3, H4, H6 and H7 were supported. However, effort expectancy and perceived risk had no significant effect on nurses’ behavioral intention to use a mobile nursing app. Therefore, H2 and H5 were not supported (Table 3).

**Table 3:** Structural model assessment and their corresponding result (*n* = 1207)

Hypothesis	Standardized Path Coefficient	*P*	Result
PE → BI (H1)	.259	<.001	Support
EE → BI (H2)	.003	.926	Not Support
SI → BI (H3)	.296	< .001	Support
FC → BI (H4)	.063	.037	Support
PR → BI (H5)	- .002	.937	Not Support
SE → BI (H6)	.344	< .001	Support
PI→BI (H7)	.091	.001	Support

## Discussion

The results of this study showed that nurses’ behavioral intention to use a mobile nursing app was positively affected by performance expectancy. If a mobile nursing app can advance nursing knowledge and is convenient for monitoring patients and sharing data, nurses will probably be more willing to use it. This was the same as the results of previous studies on the influencing factors of medical staff’s intention to use medical information technology [58, 67]. A literature review on the influencing factors of nurses’ intention to use medical information technology pointed out that perceived functional benefits of medical information technology was the direct predictors of nurses’ intention to use medical information technology [68]. At the same time, it was pointed out that the functional benefits of medical information technology mainly include the improvement of nursing quality, nursing work efficiency and patient safety [68]. Therefore, medical institutions should conduct an in-depth analysis of nurses’ expectations for a mobile nursing app to ensure that their work needs are met. In this way, the benefits of a mobile nursing app can be given full play, and nurses are more willing to rely on customized a mobile nursing app [69, 70].

In the original UTAUT model, effort expectancy affected behavioral intention [20]; however, our research does not support this relationship. Effort expectancy is not an influential factor for the behavioral intention of a mobile nursing app by nurses in Chinese hospitals. Strudwick [71] pointed out in the literature review that effort expectancy of information technology in m-Health by nurses may not directly affect nurses’ behavioral intention to use medical information technology. Shiferaw et al. [69] used the UTAUT model to explore the influencing factors of medical staff’s behavioral intention and behavior to use the electronic medical record system. The results indicated that effort expectancy did not affect the behavioral intention of medical staff to use the electronic medical record system, but effort expectancy positively affected the behavior of medical staff to use the electronic medical record system. Maillet et al. [67] explored the influencing factors of 616 nurses’ satisfaction with the use of the electronic medical record system based on UTAUT model, and the study indicated that effort expectancy positively affected nurses’ satisfaction with the use of the electronic medical record system. Therefore, effort expectancy of a mobile nursing app by nurses does not necessarily affect nurses’ behavioral intention to use a mobile nursing app. However, effort expectancy of a mobile nursing app by nurses may have a impact on nurses’ behavior or satisfaction with the use of a mobile nursing app. Therefore, in this study, although effort expectancy of nurses on a mobile nursing app did not affect their intention to use, the psychological activities of nurses when they actually use a mobile nursing app should be fully considered. It is particularly important to reduce the negative emotions and frustration caused by the actual use of a mobile nursing app by nurses. It is suggested that nursing managers urge the software developers to simplify the operation difficulty of a mobile nursing app, ensure that a mobile nursing app is easy to use, thus reducing the frustration encountered by nurses in the real process of using a mobile nursing app.

The results of this study show that social influence had a positive impact on nurses’ intention to use a mobile nursing app. Ma et al. [72] used UTAUT model to explore the influencing factors of 194 doctors’ intention to use online medical service platform and their use behavior in Beijing, China. The results showed that Beijing doctors’ intention to use online medical service platform was positively affected by performance expectancy, effort expectancy, social influence and facilitating conditions, among which the social influence has the greatest influence on the intention to use online medical service platform. Through interviews with some investigated doctors, the behavior that long-time using online medical service platforms of doctors was mostly a group behavior in the unit of the department [72]. Doctors had herd mentality and herd behavior when using online medical service platform. Most of them were influenced by their colleagues and the overall environment of the hospital so as to use online medical service platform for a long time. Nurses may also have a certain herd mentality in their intention to use a mobile nursing app. Therefore, nursing management and the government can strengthen the publicity and guidance of a mobile nursing app, a new type of nursing service, through multiple channels, so as to improve the intention of nurses to use a mobile nursing app.

The results of this study showed that nurses’ intention to use a mobile nursing app was positively affected by facilitating conditions. Our findings are consistent with previous research about behavioral intention in m-Health [43, 58, 73]. To improve the supporting equipment required by the use environment of a mobile nursing app, therefore, relevant equipment for using a mobile nursing app should be customized according to the working environment of nursing staff.

Our study found that the behavioral intention of nurses to use a mobile nursing app was not affected by perceived risk. The possible reason may be that the use of smart phones had been normalized and popularized among Chinese people, which made people generally insensitive to the security of smart phone software. Ma et al. [72] pointed out in their study that Beijing doctors’ behavioral intention and behavior to use online medical service platform were not affected by perceived risk. It was pointed out that most doctors has certain concerns about the security of the online medical service platform, the authenticity of the information released and the protection of personal privacy [72]. But because Beijing doctors perceived that the use of new internet technologies was the general trend. Therefore, perceived risk of Beijing doctors to the online medical service platform had no influence on the intention and behavior to use the online medical service platform [72].

The results of our study found that self-efficacy of a mobile nursing app of 1207 nurses was at a medium and high level. Also found that the behavioral intention of nurses to use a mobile nursing app was positively affected by self-efficacy. This may be because the stronger the ability of nurses to use smart phone applications, the more confident they are to master the use of a mobile nursing app [68]. Ifinedo [25] pointed out that the computer self-efficacy of Canadian nurses had a positive effect on a nursing information system. Previous studies had confirmed self-efficacy played an important role in nurses’ intention to use information technology [56, 69, 74]. In addition, this study also found that among the influencing factors of nurses’ intention to use a mobile nursing app, self-efficacy was the strongest influencing factor. Therefore, it is very important to train nurses’ mobile application operation ability to promote a mobile nursing APP. The training aims to improve nurses’ awareness of software exploration, information awareness and information literacy.

The results of our study showed that the behavioral intention of nurses to use a mobile nursing app was positively influenced by perceived incentives, and the effect value of perceived incentives on the behavioral intention was low. Tsai et al. [62] pointed out that due to the immaturity of electronic medical record system in Taiwan, doctors did not only use the electronic medical record system but also use paper medical records when recording patients’ conditions. This dual recording process increaseed the workload of doctors and reduced the time doctors spend treating patients. This had led to doctors’ dissatisfaction with the use of the electronic medical record system. The United States has introduced a HITECH law that provides incentives for doctors who use EHCR. Doctors’ use of EHCR increased after they were given mandatory incentives in 2012 [75]. In this regard, the construction of nursing informatization in China also needs the cooperation between government departments and social resources. Government departments should establish a reasonable incentive and constraint mechanism for the promotion and application of a mobile nursing app. Attract high-quality social resources to participate in the construction of a mobile nursing app projects in order to promote the further development of China’s nursing information.

### Limitations

There were several limitations to this study. First, participants were only recruited from Shandong, China. Further research should compare the differences in nurses’ behavioral intention among several provinces in China. Second, we only focused on behavioral intention concerning a mobile nursing app. Future research could test the actual usage behavior for mobile nursing apps and investigate the related factors.

## Conclusion

Our study examined what factors influence nurses’ behavioral intention of a mobile nursing app. As expected, performance expectancy, social influence, facilitating condition, self-efficacy and perceived incentives had a positive significant effect on nurses’ behavioral intention to use a mobile nursing app. With 70.2% of the variance in behavioral intention to use a mobile nursing app explained by this model, it could be helpful for potential adopters, and further investigation should test the actual usage behavior for mobile nursing apps and investigate the related factors.

## Supplementary Information

**Additional file 1 Appendix**: Questionnaire items.

## Data Availability

All data generated or analysed during this study are included in this published article.
