# Supplementary material for: Determinants of the behavioral intention to use a mobile nursing application by nurses in China
Source: BMC Health Serv Res. 2021 Mar 12;21:228. doi: 10.1186/s12913-021-06244-3 (PMC7953719; doi:10.1186/s12913-021-06244-3)
Supplement: Supplementary file 1 — Additional file 1 Appendix: Questionnaire items. [file 12913_2021_6244_MOESM1_ESM.docx]

**Appendix: Questionnaire items**

*Performance Expectancy*

PE1. I think a mobile nursing app will improve my nursing knowledge.

PE2. A mobile nursing app will be very convenient to monitor the patient's condition or health status.

PE3. A mobile nursing app will facilitate the information sharing of patients' health management process.

*Eﬀort Expectancy*

EE1. I think it will be easy to learn to use a mobile nursing app.

EE2. I think it will be easy to operate a mobile nursing app.

EE3. It's easy for me to get the information I want in a mobile nursing app.

*Social Inﬂuence*

SI1. The attitude of the hospital management will affect my willingness to use a mobile nursing app.

SI2. The government's new policy on mobile health will encourage me to try a mobile nursing app.

SI3. The advertisement of mobile medical software will prompt me to try to use a mobile nursing app.

*Facilitating Condition*

FC1. The reaction speed of a mobile nursing app is very important to me.

FC2. It is very important for me to have a reasonable page layout of a mobile nursing app.

FC3. It is very important for me to be trained on the technology use of a mobile nursing app.

*Perceived Risk*

PR1. I think a mobile nursing app may leak personal privacy.

PR2. I think a mobile nursing app may be bundled with malicious appliacation, which will cause unnecessary trouble.

PR3. I think the existing a mobile nursing app related laws are not sound, which leads to my rights cannot be fully protected.

*Self-Efficacy*

SE1. I can skillfully use various applications in my mobile phone.

SE2. After downloading the new application, I can quickly learn how to use it.

SE3. It is within my ability to use mobile applications.

*perceived incentives*

PI1. I think in the current work environment, the use of a mobile nursing app need incentives.

PI2. I think using a mobile nursing app can give me some extra pay.

PI3. In my opinion, promoting the use of a mobile nursing app in the current working environment requires material incentives.

*Behavioral Intention*

BI1. I am willing to use a mobile nursing app to assist my nursing work.

BI2. I am willing to manage patients on a mobile nursing app.

BI3. I would like to recommend my colleagues to use a mobile nursing app.
